# Fracture care using percutaneously applied titanium mesh cages (OsseoFix®) for unstable osteoporotic thoracolumbar burst fractures is able to reduce cement-associated complications—results after 12 months

**DOI:** 10.1186/s13018-015-0322-5

**Published:** 2015-11-14

**Authors:** Stephan Albrecht Ender, Anica Eschler, Michaela Ender, Harry Rudolf Merk, Ralph Kayser

**Affiliations:** Department of Orthopaedics and Orthopaedic Surgery, University Medicine Greifswald, Ferdinand-Sauerbruch Straße, 17475 Greifswald, Germany; Department of Trauma, Hand and Reconstructive Surgery, University of Rostock, Medical Center, Schillingallee 35, 18057 Rostock, Germany; Department of Diagnostic Radiology and Neuroradiologie, University Medicine Greifswald, Ferdinand-Sauerbruch Straße, 17475 Greifswald, Germany; Department of Orthopaedics, Trauma and Reconstructive Surgery, Charité University Medicine Berlin, Campus Benjamin Franklin, Hindenburgdamm 30, 12203 Berlin, Germany

**Keywords:** Osteoporosis, Incomplete vertebral burst fracture, OsseoFix, Minimally invasive, Titanium mesh cage

## Abstract

**Background:**

Despite the known demographic shift with expected doubled rate of vertebral body fractures by the year 2050, a standardized treatment concept for traumatic and osteoporotic incomplete burst fracture of the truncal spine does not exist. This study aims to determine whether minimally invasive fracture care for incomplete osteoporotic thoracolumbar burst fractures using intravertebral expandable titanium mesh cages is a suitable procedure and may provide improved safety in terms of cement-associated complications in comparison to kyphoplasty procedure.

**Methods:**

In 2011/2012, 15 patients (10 women, 5 men; mean age 77) with 15 incomplete osteoporotic thoracolumbar burst fractures (T10 to L4) were stabilized using intravertebral expandable titanium mesh cages (OsseoFix®) as part of a prospective study. X-ray, MRI and bone density measurements (DXA) were performed preinterventionally. The clinical and radiological results were evaluated preoperatively, postoperatively and after 12 months according to the visual analogue scale (VAS), the Oswestry Disability Index (ODI), X-ray (Beck Index, Cobb angle) and CT analyses. Wilcoxon rank sum test, sign test and Fischer’s exact test were used for statistical evaluation.

**Results:**

A significant reduction in pain intensity (VAS) from preoperative 8.0 to 1.6 after 12 months and significant improvement in activity level (ODI) from preoperative 79.0 to 30.5 % after 12 months were revealed. Radiologically, the mean kyphotic angle according to Cobb showed significant improvements from preoperative 9.1° to 8.0° after 12 months. A vertebral body subsidence was revealed in only one case (6.7 %). No changes in the position of the posterior wall were revealed. No cement leakage or perioperative complications were seen.

**Conclusion:**

As a safe and effective procedure, the use of intravertebral expandable titanium mesh cages presents a valuable alternative to usual intravertebral stabilization procedures for incomplete osteoporotic burst fractures and bears the potential to reduce cement-associated complications.

**Trial registration:**

German Clinical Trials Register (DKRS) DRKS00008833.

## Background

The demographic development of Western Europe and North America towards an older society accounts for an increase in the incidence of osteoporosis and people suffering from osteoporotic vertebral body fractures [1–6]. Owing to this demographic shift, double the rate of vertebral body fractures is expected by the year 2050 [3]. More than 320,000 vertebral body fractures of osteoporotic origin are newly diagnosed in Europe every year, the majority of these involving fractures of AO type A1 (AO classification [7]). To a lesser extent, fractures involving the posterior wall of the vertebral body, AO type A3 (incomplete burst fracture), can regularly be found, which are infrequently accompanied by neurological impairments [8–10].

A standardized treatment concept for traumatic and osteoporotic incomplete burst fracture of the truncal spine does not exist either internationally or nationally in Germany [10, 11]. There is a wide spectrum of conservative and operative therapeutic approaches [1, 10, 11]. Conservative treatment is only indicated without significant misalignment, spinal stenosis or neurological deficit [10–13]. Operative stabilization is recommended if there is an increase in kyphotic misalignment, dislocation of the vertebral body posterior wall fragment as well as immobilization due to pain after conservative therapy has been exhausted [1, 10, 14, 15]. The type of surgical approach to use is discussed with controversy: Minimally invasive intravertebral stabilization procedures, for example kyphoplasty; dorsal stabilization procedures (internal fixator), applicable with cement-augmented pedicle screw fixation; and the combination of both procedures (hybrid treatment) may be used [1, 11, 14, 15]. Particularly in older people, bone quality and general state of health are considered in the choice of therapy options, and minimally invasive treatment options are included [14, 15]. Kyphoplasty is an established and readily used minimally invasive procedure for intravertebral stabilization in this respect [11, 14–19]; however, vertebral body fractures involving the posterior wall are limited to treatment with kyphoplasty [1, 20, 21] with possible leakage of bone cement into the epidural space as a known special complication of kyphoplasty [11, 18, 22, 23]. Therefore, suitable treatment alternatives are sought. Since 2009, the expandable titanium mesh cage (OsseoFix®) has been available as an alternative to kyphoplasty for minimally invasive vertebral body stabilization in osteoporotic thoracolumbar vertebral body fractures [24]. The purpose of this study was the evaluation of incomplete osteoporotic thoracolumbar vertebral body burst fractures after stabilization using intravertebral expandable titanium mesh cages. An analysis of our clinical and radiological results has been undertaken following a 12-month period in 15 patients with 15 incomplete thoracolumbar burst fractures.

## Methods

### Patients

**Fig. 1:**
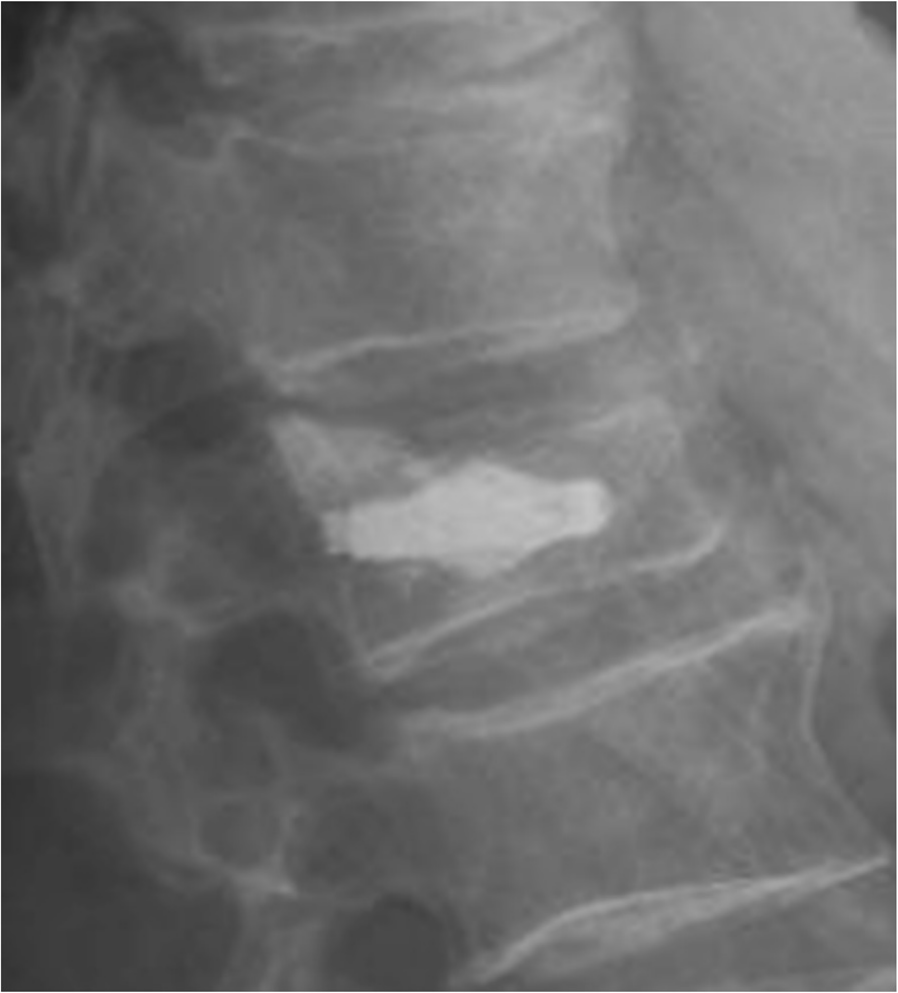
Vertebral body stabilization using the cement-augmented titanium mesh cages

As part of a prospective study, 15 symptomatic osteoporotic incomplete vertebral body burst fractures (AO type A3) in 15 patients (10 women, 5 men; mean age 77 years, min. 55, max. 89) were stabilized operatively in 2011 and 2012. In all cases, the vertebral body stabilization was carried out with solitary percutaneous implantation of two cement-augmented expandable titanium mesh cages (OsseoFix®, Alphatec Spine Inc., Carlsbad, CA, USA; Fig. 1).

The study was approved by the ethical committee at the University Medicine Greifswald under the reference number BB 132/12 and is in accordance with the Declaration of Helsinki. Informed consent was given by all patients.

Preoperatively, the mean duration of symptoms was 8.3 weeks (min. 3, max. 13). A conservative therapeutic approach prior to the operation had not resulted in any significant relief of symptoms.

A total of 11 lumbar vertebrae were stabilized (*n* = 5 L1, *n* = 3 L2, *n* = 1 L3, *n* = 2 L4) along with 4 thoracic vertebrae (*n* = 1 T10, *n* = 1 T11, *n* = 2 T12). Titanium mesh cage implantation was carried out bipedicular in all cases. An additional stabilization of an adjacent vertebral body compression fracture was carried out in one patient, analogously using the intravertebral expandable titanium mesh cages (A3 L2 and A1 L3).

*Inclusion criteria* were a symptomatic recent lumbar and/or thoracic osteoporotic incomplete burst fracture (AO type A3) and exhaustive conservative therapy.

*Exclusion criteria* were symptoms of neurological deficits, involvement of the posterior wall with relevant stenosis of the spinal canal and a known allergy to the ingredients of the OsseoFix® system and/or the bone cement.

Preoperatively, a clinical examination including and evaluation of the medical history, assessment of the pain intensity (visual pain analogue scale (VAS)) and the activity level (Oswestry Disability Index (ODI)) [25] were evaluated, and diagnostic X-rays of the region in standing position in two planes, an MRI (T1w and T2w sequences including fat suppressed sequences) and bone density measurement (DXA) in the area of the lumbar spine (L1 to L4) and the proximal femur (Lunar Prodigy Advance, General Electric) were carried out. None of the patients in this study had contraindication to MRI; therefore, MRI was performed in all cases. The preoperative MRI showed a high-intensity signal on the STIR sequence as a sign of a fresh vertebral fracture in all cases.

**Fig. 2:**
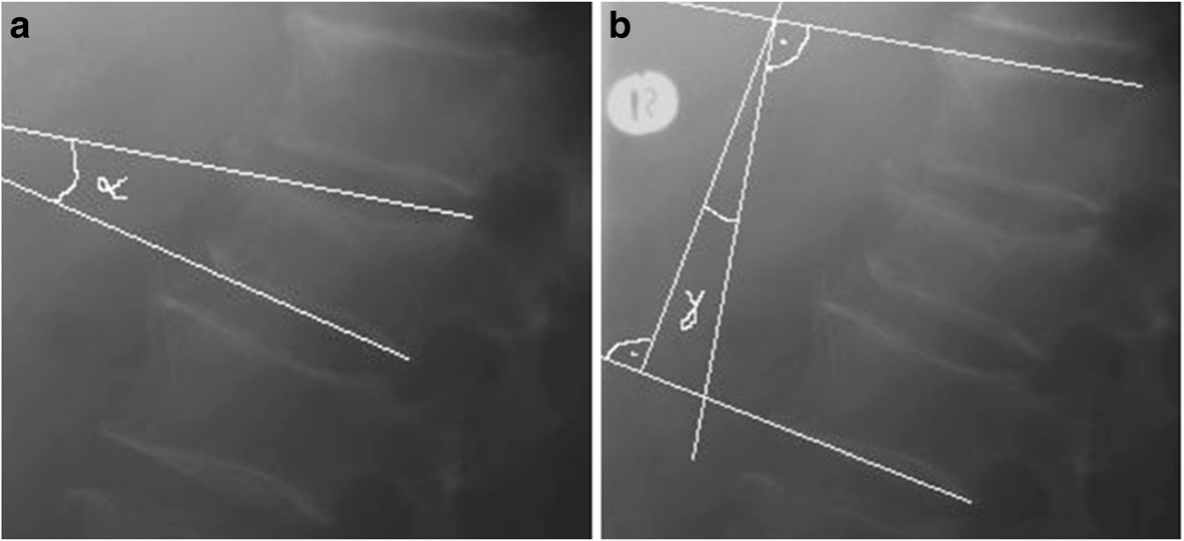
**a**, **b** Representation of the determination of the α-kyphotic angle and γ-kyphotic angle

Clinical and radiological follow-up was carried out 3 days postoperatively and after 12 months (min. 12, max. 15). The *clinical evaluation* included re-evaluation of the VAS and the ODI. The *radiological evaluation* was carried out by means of diagnostic X-ray of the thoracolumbar region in the standing position in two planes and postoperative CT scans. For quantitative evaluation of the vertebral body deformity, the Beck Index [26] (anterior vertebral height to posterior vertebral height), the vertebral kyphotic angle (α-angle) and the regional kyphotic angle (γ-angle) [27] according to Cobb were determined (Fig. 2a, b).

Definition of a kyphotic angle in case of underlying kyphosis is a positive sign and in case of underlying lordosis a negative sign [28]. The radiological follow-up included an evaluation regarding subsidence and changes to the posterior wall of the stabilized vertebral body, cement exudate, implant dislocation as well as occurrence of adjacent fractures. Whether a relationship exists between changes in the sagittal spinal profile and the activity level (ODI) was also determined.

The *statistical evaluation* was carried out with statistical software SAS® Version 9.1 with the Wilcoxon rank sum test for continuous variables (α- and γ-kyphotic angles) and with the sign test for non-continuous variables (ODI and VAS). The evaluation with regard to a correlation between postoperative kyphotic angles according to Cobb and ODI was carried out with Fischer’s exact test. The results were determined in mean and standard deviations (SD). A probability of *p* < 0.05 was stipulated to reject the null hypothesis and to show a statistically significant difference.

### Technique/application

**Fig. 3:**
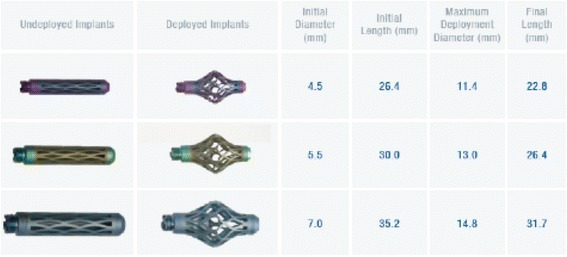
Titanium mesh cages (OsseoFix®) with technical specifications [picture copyright: Alphatec Spine Inc., Carlsbad, CA, USA]

Vertebral body stabilization using the titanium mesh cages in the previously described manner was carried out in all cases [29]. At present, solitary use of the intravertebral expandable titanium mesh cages (OsseoFix®) using only bone cement is authorized. The mesh cages consist of a combination of titanium alloy (Ti-6Al-4V, ASTM F136) with pure titanium (Ti-CP2, ASTM F67) and are specified for use in the area of T1 to L5. Three cage sizes are available for selection, which have an original diameter (unexpanded) of 4.5, 5.5 and 7.0 mm (Fig. 3).

In 12 vertebral bodies, titanium mesh cages of the original size (not expanded) of 4.5 mm (T10 to L4) were used, and in three vertebral bodies, size 5.5 mm (L2 to L4) was used; in all cases, bipedicular implantation of the same size was carried out. An average of 0.64 mL of bone cement was applied per cage (SD ±0.11, min. 0.5, max. 0.9), where more cement was applied to the larger implants.

The average operation duration per vertebral body was 54 min (SD ±8.4, min. 41, max. 81), and a fluoroscopy duration of 1.32 min (SD ±0.30, min. 0.43, max. 2.97) with a cumulative radiation dose of 17.9 mGy (SD ±2.4, min. 9.4, max. 35.2) was necessary.

The operations were always carried out under intubation anesthesia, and the patients received a perioperative single shot of IV antibiotic (1.5 g cefuroxime or in the case of existing allergy 600 mg clindamycin). Intraoperatively, a uniplanar fluoroscopy system (Veradius, Philips, Netherlands) with a dual monitor was used.

Postoperatively, mobilization was carried out from the first postoperative day according to the patient’s pain threshold under the supervision of a physiotherapist along with implementation of physical therapy for strengthening of the spinal stabilization musculature in the course of further treatment. In all patients, postoperative thromboembolism prophylaxis with a low molecular weight heparin derivative was implemented. Pre-existing pain medication was continued postoperatively and reduced over the course of time.

In all cases, existing special osteoporosis medication was to be continued (21 % of the patients) or an oral medication with a bisphosphonate was to be begun (79 %).

## Results

### Clinical evaluation

Fifteen patients (15 treated vertebral body fractures) were evaluated preoperatively, postoperatively and after a follow-up period of 12 months.

There was a significant improvement (*p* < 0.001) in the mean VAS and the ODI after 12 months. The mean preoperative VAS was 8.0 points (SD ±1.9, min. 6, max. 10), postoperative 2.7 points (SD ±1.4, min. 0, max. 5) and after 12 months 1.6 points (SD ±0.90, min. 0, max. 3). The mean preoperative ODI accounted for 79.0 % (SD ±3.5, min. 62, max. 76), the postoperative ODI for 31.6 % (SD ±3.9, min. 28, max. 48) and after 12 months 30.5 % (SD ±3.2, min. 28, max. 42) were evaluated (Table 1).

**Table 1:** Changes in ODI and VAS preoperatively, 3 days postoperative and at 12 months follow-up

Clinical evaluation	Average value	Average value	Average value	*p* value
	Preop.	3 days postop.	After 12 months	Preop.-12 months
VAS	8.0	2.7	1.6	<0.001
ODI	79.0 %	31.6 %	30.5 %	<0.001

### Radiological evaluation

The mean Beck Index changed from preoperative 0.71 (SD ±0.11, min. 0.42, max. 0.88) to postoperative 0.74 (SD ±0.12, min. 0.5, max. 0.98). During the follow-up period, the mean Beck Index of 0.74 (SD ±0.13, min. 0.5, max. 0.98) was constant. The changes in the mean vertebral kyphotic angle (α-angle) and the mean kyphotic angle according to Cobb (γ-angle) are summarized in Table 2.

**Table 2:** Changes in sagittal spine alignment—preoperative, 3 days postoperative and after 12 months follow-up

Sagittal spine alignment	Average value	Average value	Average value	*p* value
	Preop.	3 days postop.	After 12 months	Preop.–12 months
Vertebral kyphotic angle (α-angle)	10.2°	9.5°	9.6°	<0.05
	(SD ±4.2, min. 3.9, max. 21.9)	(SD ±3.9, min. 3.7, max. 20.2)	(SD ±4.1, min. 3.6, max. 20.1)	
Kyphotic angle according to Cobb (γ-angle)	9.1°	8.0°	8.0°	<0.05
	(SD ±11.3, min. −20.0, max. 28.0)	(SD ±11.6, min. −24.0, max. 32.2)	(SD ±11.6, min. −24.0, max. 32.0)	

No changes in the position of the posterior wall and no cement leakage were seen.

**Fig. 4:**
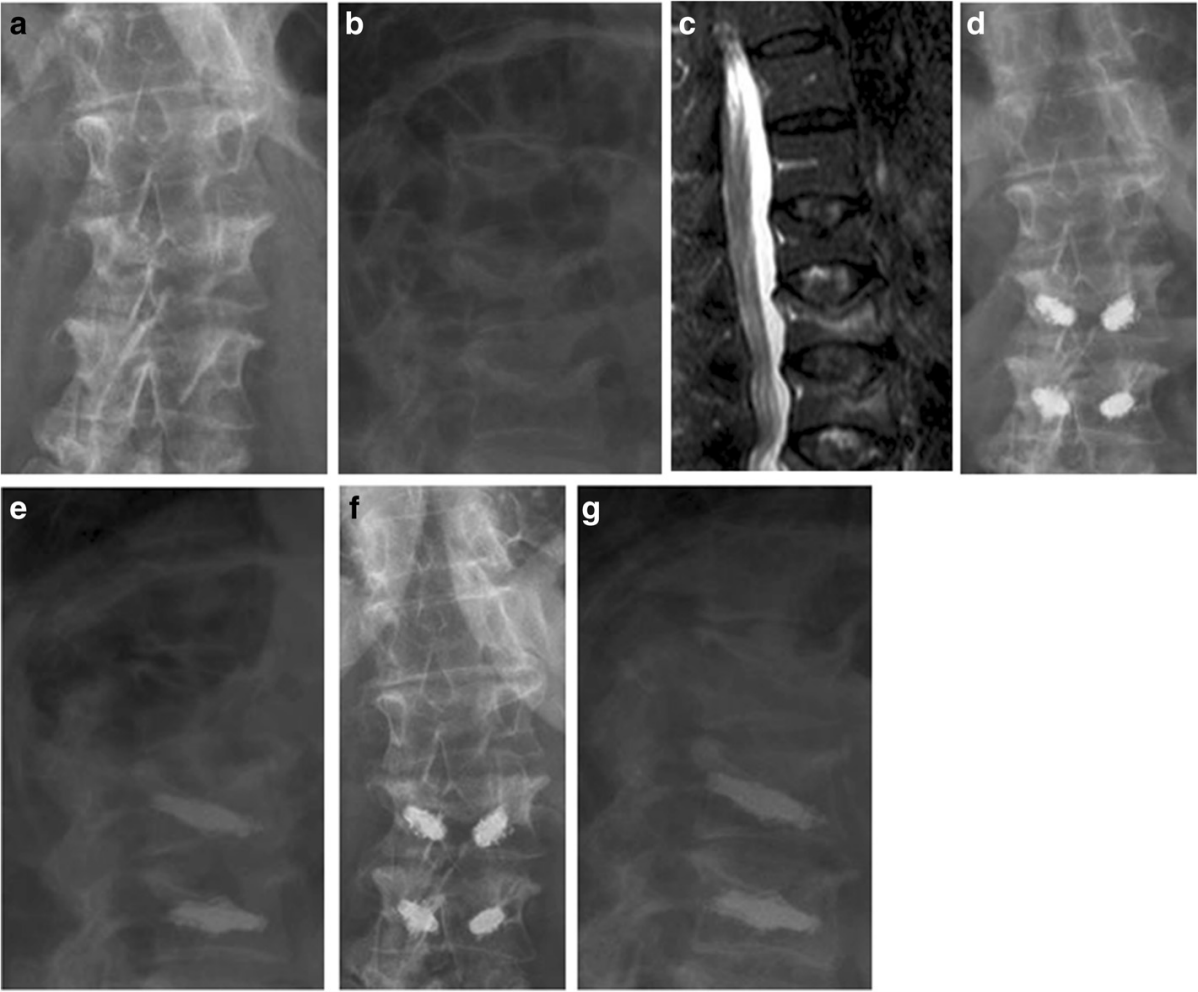
**a**–**g** Clinical case with X-ray imaging and MRI. Legend: Preoperative (**a**–**c**), postoperative (**d**, **e**) and X-ray imaging as well as MRI after 12 months (**f**, **g**) of an osteoporotic incomplete L2 burst fracture and L3 compression fracture without involvement of the posterior wall and stabilization using two cement-augmented titanium mesh cages (preoperative VAS 10.0, ODI 70.0 %; 12 months postoperative VAS 2.0, ODI 34.0 %)

The preoperative bone density measurement (DXA) revealed a mean BMD of 0.7654 g/cm^2^ (SD ±0.08, min. 0.91, max. 0.52), a mean *T*-score of −3.6 (SD ±0.68, min. −2.0, max. −5.4) and a mean *Z*-score of −1.8 (SD ±0.68, min. −0.9, max. −3.9). Figure 4 shows radiological progress diagnostics.

### General complications

No perioperative complications and no further postoperative complications (up to 3 months after intervention) in terms of bleeding, wound healing impairment, infection, phlebothrombosis and pulmonary embolism occurred.

### Special complications

No neurological complications arose. In one case (6.7 %), a slight subsidence of vertebral height was revealed during the follow-up period. For this specific patient, the Beck Index changed from 1.0 postoperative to 0.96 after 12 months and the kyphotic angle according to Cobb from 11.0° to 13.0°. The VAS score (2.0) remained unchanged during the course.

## Discussion

Due to the demographic change in Western Europe and North America with an increasing incidence of vertebral body fractures of osteoporotic origin, the number of incomplete burst fractures with involvement of the posterior wall, AO type A3, is also on a rise [1–6, 8–10]. After conservative therapy has been exhausted, the question of operative treatment remains as to which procedure to choose, taking into account the invasiveness and the clinical results, where minimally invasive treatment options continue to come to the forefront [14, 15]. Frequently used dorsal stabilization procedures with internal fixators are associated with a certain rate of implant failure with consequently progressive kyphotic malalignment [1, 21, 30, 31]; besides, augmented pedicel screws show elevated rates of cement leakages [1, 32]. Kyphoplasty, as a well-established minimally invasive procedure for intravertebral stabilization, is also used in the stabilization of incomplete burst fractures with good clinical results [11, 14–19]; however, leakage of bone cement into the epidural space is known as a special complication of kyphoplasty [11, 18, 22, 23]. Thus, involvement of the posterior wall is a limiting factor for the treatment of incomplete burst fractures using kyphoplasty [1, 20, 21].

With the expandable titanium mesh cages, an interesting alternative has existed since 2009 for percutaneous minimally invasive intravertebral stabilization of incomplete thoracolumbar burst fractures (AO type A3) [24]. A discussion of the results follows in regard to kyphoplasty as the current gold standard for minimally invasive intravertebral stabilization.

*The results of this study* using intravertebral expandable titanium mesh cages in a stand-alone technique reveal comparable clinical results in the first postoperative year when compared to kyphoplasty [17–19, 33]. We did not reveal any cement leakage (kyphoplasty 11 to 47 %, titanium mesh cage 0 %) and little subsidence rates (kyphoplasty 8 to 30 %, titanium mesh cage 7 %) [11, 17–19]. A significant improvement in the pain intensity (VAS) and the activity level (ODI) after 12 months was revealed for this study. In one case, we saw a slight vertebral endplate subsidence during follow-up (L2). Data with regard to a secondary loss of height are described in the literature for kyphoplasty with 0 to 50 % [17, 34, 35] (follow-up period 6 to 27 months). The rate of a secondary subsidence of 7 % in the context of vertebral body stabilization with the titanium mesh cage in the stand-alone technique is slight and, however, is adding to the fact that vertebral body reduction with the titanium mesh cage is slightly decreased when compared to kyphoplasty. The amount of vertebral height reduction, however, leads to no significant improvement in clinical condition [20], whereas a correlation between an increased risk of adjacent fractures and the degree of vertebral body reduction is to be accounted for [21, 22].

The mean kyphotic angle according to Cobb improved significantly from preoperative 9° to 8° after 12 months (*p* < 0.05). For kyphoplasty, distinctly better values are given with a mean improvement of the kyphotic angle according to Cobb from 4° to 8° [11, 33, 36, 37]. We did not find a correlation between the amount of improvement of the kyphotic angle according to Cobb and the clinical outcome (ODI) (*p* = 1.0). With the OsseoFix® system, only small amounts of bone cement are needed to fill the cavity of the spread titanium mesh cage (per titanium mesh an average of 0.65 mL). Cement leakage was not seen in the context of titanium mesh cage stabilization (cement leakage 0 %) which is likely associated with the reduced cement amounts needed. In the context of stabilization of osteoporotic incomplete vertebral body fractures with balloon kyphoplasty, cement leakage rates are given from 11 to 47 % (1 % symptomatic) [11, 17, 18, 38, 39], whereby this is noteworthy considering the proven correlation between intradisc cement leakage rates and the elevated emergence of adjacent fractures [40, 41]. From this perspective, use of the cement-augmented expandable titanium mesh cages in osteoporotic incomplete burst fractures appears to be an improved and valuable treatment option.

## Conclusions

The stabilization of symptomatic osteoporotic incomplete burst fractures with the cement-augmented expandable titanium mesh cages shows favourable clinical results within the first postoperative year. The technique shows comparable clinical results to kyphoplasty with reduced complication rates in terms of cement leakage and secondary vertebral subsidence. Despite the slightly reduced vertebral body reduction capabilities with use of the expandable titanium mesh cages, no negative influence to the clinical outcome was revealed, thereby affirming the experience previously established with kyphoplasty.

As a safe and effective procedure, the use of the expandable titanium mesh cages presents a valuable treatment alternative to the currently used intravertebral stabilization procedures for incomplete vertebral body fractures.
